# Genetic Factors of Individual Differences in Decision Making in Economic Behavior: A Japanese Twin Study using the Allais Problem

**DOI:** 10.3389/fpsyg.2015.01712

**Published:** 2015-11-13

**Authors:** Chizuru Shikishima, Kai Hiraishi, Shinji Yamagata, Juko Ando, Mitsuhiro Okada

**Affiliations:** ^1^Department of Psychology, Faculty of Liberal Arts, Teikyo UniversityTokyo, Japan; ^2^Faculty of Letters, Keio UniversityTokyo, Japan; ^3^Faculty of Arts and Science, Kyushu UniversityFukuoka, Japan

**Keywords:** decision making, Allais problem, expected utility theory, prospect theory, cognitive ability, twin, behavioral genetics

## Abstract

Why does decision making differ among individuals? People sometimes make seemingly inconsistent decisions with lower expected (monetary) utility even when objective information of probabilities and reward are provided. It is noteworthy, however, that a certain proportion of people do not provide anomalous responses, choosing the alternatives with higher expected utility, thus appearing to be more “rational.” We investigated the genetic and environmental influences on these types of individual differences in decision making using a classical Allais problem task. Participants were 1,199 Japanese adult twins aged 20–47. Univariate genetic analysis revealed that approximately a third of the Allais problem response variance was explained by genetic factors and the rest by environmental factors unique to individuals and measurement error. The environmental factor shared between families did not contribute to the variance. Subsequent multivariate genetic analysis clarified that decision making using the expected utility theory was associated with general intelligence and that the association was largely mediated by the same genetic factor. We approach the mechanism underlying two types of “rational” decision making from the perspective of genetic correlations with cognitive abilities.

## Introduction

We know that human decision making is often inconsistent and violates the rational assumptions of economics (e.g., Allais, 1953, 1979; Ellsberg, 1961). Behavioral economists argue that the traditional view of Homo economicus does not apply to human decision-making behavior (Kahneman and Tversky, 1979). However, we also know that decision making for some people appears to be more consistent and “rational” than for others. In addition, the very definition of rationality is a target of intense controversy (e.g., Gigerenzer et al., 1999; Stanovich and West, 2000). The present study focuses on individual differences in decision making in economic behavior and attempts to identify their source, taking into account individual genetic differences. In doing so, we will argue that a certain type of rational decision making is associated with cognitive abilities and that the association is genetically mediated.

In the current post-genomic era, people are more aware of the importance of genetics as the source of differences in human behavior. In fact, behavioral genetic studies worldwide have revealed that in any population subjected to study, more than half of the total variance in intelligence (e.g., Bouchard and McGue, 1981) and nearly half of the total variance in personality (e.g., Bouchard and Loehlin, 2001) are explained by individual differences in genes. With such a high genetic effect, human psychological traits, particularly intelligence and personality, are known to be relatively stable across age within the individual.

Decision making is no exception. Using the twin method, which compares the similarity of responses between identical twin pairs and fraternal twin pairs, substantial genetic influences on imaginary decision-making tasks in the field of behavioral economics, and real-life economic decision-making behavior, have been reported in recent behavioral genetic studies. For example, a third of the variance in investment behavior, such as participation in the stock market and asset allocation, can be explained by a genetic factor (Barnea et al., 2010). Savings behavior is 35% genetic (Cronqvist and Siegel, 2013) and investment portfolio risk behavior 25% (Cesarini et al., 2010).

Behavioral genetic studies on economic games in laboratory experiments have produced similar results. For instance, Cesarini et al. (2008) conducted trust game experiments in two countries and revealed that the heritability of trust was 32% for the Swedish study and 17% for the USA study; heritabilities of trustworthiness were 20 and 10%, respectively. Wallace et al. (2007) estimated the heritability of responses on an ultimatum game. They revealed that the minimum acceptance threshold, the smallest amount of money below which one will reject an offer of money, is 42% genetic. A review of the genetic effect on economic games reported that strategies and fundamental economic preference for trust, dictator, and ultimatum games were all genetic, with heritabilities ranging from 18 to 42% (Cesarini et al., 2009a). However, genetic effects are not always a significant source of individual differences in decision making in economic behavior. Simonson and Sela (2011) found no genetic effects for some features of consumer judgment and choice.

Although there are some exceptions or differences in degree, ample empirical evidence suggests a genetic foundation for individual differences in decision making in economic behavior. Genes do matter for human decision making. It is unreasonable to assume that genes contribute to specific decisions or choices; however, we can assume that decision making is genetic because its mechanism reflects some intrinsic, human genetic predispositions.

Behavioral economic studies occasionally refer to preferences as individual innate stable dispositions underlying decision-making behavior and attribute genetic influences on decision making to genetic factors underlying personal preferences. In one study examining the source of investment behavior, the genetics of asset allocation behavior were interpreted as deriving from the genetics of risk preferences (Barnea et al., 2010). Giving and risk-taking preferences elicited by several economic games were identified as genetic with a heritability of around 20% (Cesarini et al., 2009b). A large genetic effect was also found for economic risk-taking preferences across economic decision-making tasks (Zyphur et al., 2009). It has been proposed that heritable individual differences in “prudence” are an underlying common factor in some features of consumer judgment and choice (Simonson and Sela, 2011).

In the present study, we assume that cognitive ability is another predisposition that underlies decision making in economic behavior. Under the presumption that people with different general cognitive ability levels should make different decisions, Frederick (2005) demonstrated that decision making with respect to time preferences and risk preferences was associated with cognitive ability. In fact, Yamagishi et al. (2014) revealed that those who make economically rational choices, earning more money while disregarding their partner’s earnings on economic games, had better scores on an IQ test. Cesarini et al. (2012) examined the association between behavioral anomalies and cognitive abilities. They found that some individual differences in anomalies (illusion of control, insensitivity to sample size, and representativeness) were correlated with cognitive ability as a result of a common genetic basis, not because of a common environmental function.

Considering this background, we predict that human decision making in economic behavior is influenced by genetics and that the genetic factor is at least in part derived from genetic influences on cognitive ability. To test these hypotheses, it is necessary to empirically show that the two observed variables of decision making and cognitive ability are correlated at the genetic factor level.

To achieve this goal, we employed the twin method, the most commonly used approach in behavior genetics, and measured both cognitive ability and decision making from the same twins. Bivariate genetic analysis with behavior genetic designs enables us to examine the extent to which genetic and environmental sources mediate the phenotypic covariance between the two variables.

To classify individual decision making into specific patterns, we employed the Allais (1953, 1979) problem. The Allais problem is the earliest and best-known paradox frequently introduced as a human decision-making task that demonstrates the violation of the axioms of expected utility theory (EUT). The problem consists of two questions. In the first question, the respondents are required to choose which they prefer in a lottery (gamble) between the options A and B. The options are depicted as follows:

Option A: One million dollars for sure

Option B: 89% probability of 1 million dollars

                     10% probability of 5 million dollars

                     1% probability of nothing.

Then, in the second question, the respondents are again required to choose which they prefer in a lottery (gamble) between the options C and D. The options are described as follows:

Option C: 11% probability of 1 million dollars

                     89% probability of nothing

Option D: 10% probability of 5 million dollars

                     90% probability of nothing.

Allais found that most people choose A from the first question and D from the second question (AD). Although the outcome of the 89% slice of probability is the same in A and B as it is in C and D, choosing A rather than B on the one hand, and choosing D rather than C on the other hand is conflicting; Allais calls this a paradox.

The Allais problem has been administered under many different conditions (e.g., Slovic and Tversky, 1974; Kahneman and Tversky, 1979; Camerer, 1989; Conlisk, 1989; Wu and Gonzalez, 1998; Birnbaum, 2004; Stanovich and West, 2008; Huck and Müller, 2012), and the results repeatedly demonstrate the paradox. For instance, a study with American university students demonstrated that the most frequently chosen combination pattern was AD (43.6%), followed by BD (41.9%), then AC (7.6%), and BC, which was the least frequently chosen (6.8%; Conlisk, 1989). More recently, another study using an internet survey with a representative sample of the Dutch population over 15 years of age found the distribution of frequency ratio to be 33.9% for AD, 30.2% for BD, 20.4% for AC, and 15.5% for BC, when the currency of dollars in the original Allais problem was replaced with Euros (Huck and Müller, 2012).

It is noteworthy that what constitutes the “rational” choice on the Allais problem is not necessarily straightforward. As mentioned above, the combination of BD is in accordance with the EUT; thus appears to be rational. However, selecting AC can also be considered rational if we take into account the respondent’s preference for more certain reward rather than larger reward; the probability of gaining some money is higher for A than for B in the first choice, and it is higher for C than for D in the second choice. In addition, even the AD choice, the choice that Allais called the paradox, can be considered rational from the perspective of the prospect theory (Kahneman and Tversky, 1979). The only choice for which no explanation can be provided is the BC choice.

The fact that no study has found robust correlations between the Allais problem and cognitive ability (e.g., Slovic and Tversky, 1974; Stanovich and West, 2000, 2008) may reflect this ambiguity regarding the problem’s rationality. It may be the case that one rational response pattern (e.g., BD choice) reflects the person’s cognitive ability while the others do not. We need to examine the relationships between each of the Allais problem response patterns and cognitive ability separately.

Additionally, it is also possible that the lack of robust findings is a product of biased samples and inadequate indices of general cognitive ability. For instance, using university students and their SAT scores, Stanovich and West (2008) found no significant associations between the response to the Allais problem and cognitive ability, and argued that the correlation between the two is elusive. Looking at the data closely, however, their results suggest that higher cognitive ability individuals (the high-SAT group) made more EUT compatible choices (choosing D instead of C in the second question). In fact, the statistical analysis approached significance (*p* = 0.073).

The association between education and the response pattern of the Allais problem also appears to be unclear. Huck and Müller (2012), elucidating that earlier research on the Allais problem has been carried out solely using student samples, examined demographic characteristics for those who violated the EUT using a large representative sample. They showed that although no difference was found in education level among the response patterns in the original version of the Allais problem where very high payoffs were hypothesized, higher education had an effect on reducing the violation of EUT in the revised version with downscaled payoffs (1 and 5 Euro instead of 1 and 5 million Euro; Huck and Müller, 2012).

Given the current state of the field, we decided to explore in depth the relationships between the Allais problem response patterns and general cognitive ability using samples with large variation in cognitive ability and valid cognitive ability tests as general intelligence indices.

To investigate logical nature of rationality, it is necessary to examine the relationships between the Allais problem response patterns and basic logical inference ability. As a basic logical task, it would be natural to use the syllogism-solving task, in which respondents are asked to determine if, under given assumptions, a conclusion is logically inferred, rather than just consistent with an assumption. The syllogism-solving task is a natural choice because (i) it provides the most traditional normative model of logical inference (dating back to Aristotle in the fourth century BC); (ii) it has been widely used for many decades by experimental psychologists for the study of logical inferences; (iii) it is still considered to constitute the core part of logical reasoning with ordinary language (see, Mineshima et al., 2012).

We examined the distribution of response patterns to the Allais problem and confirmed whether the paradox replicated in the West was also apparent in a Japanese sample. To the authors’ knowledge, there is no report on the distribution of Japanese response patterns for the Allais problem. Univariate genetic analyses with a behavioral genetic approach were conducted on each response pattern, to clarify the genetic and environmental effects that contributed to the decision making. Subsequently, we investigated the association between response patterns and cognitive ability. The sample in the present study comprised twins with ample variation in educational backgrounds. The cognitive ability tests we used included ones that measured not only general intelligence but also syllogistic inference ability with high reliability and validity. The mean level of performance on each cognitive ability test was compared among the response patterns. Bivariate genetic analyses were conducted to clarify the genetic and environmental overlap between cognitive ability and decision making.

## Materials and Methods

### Participants

The participants were 1,199 (886 female and 313 male) Japanese adult twins registered with the Keio Twin Study (KTS; Shikishima et al., 2006; Ando et al., 2013). The KTS recruited 14–30-years-old twin participants through population-based registries in some parts of the Tokyo area from 1998 to 2011. After recruitment, the registrants were assessed by questionnaires delivered via mail surveys and by cognitive ability tests or physical measurements administered at the university. The Allais problem was included in the comprehensive postal survey conducted by the KTS in 2012. The age of the survey respondents ranged from 20 to 47, with a mean of 26.57 and standardized deviation of 4.94. The distribution of respondents’ educational attainment level was junior high school 1%, high school 10%, junior/technical/vocational college 29%, university 54%, and graduate school 6%.

The zygosity of each same-sex twin pair was initially diagnosed by a three-items questionnaire based on physical resemblance (Ooki et al., 1990). Gene polymorphisms were examined in nearly half the pairs. It was confirmed that 93.3% of these DNA-based diagnoses were in agreement with the initial questionnaire-based diagnoses. As a result, the effective number of complete twin pairs who responded to the Allais problem was broken down as follows: 291 female monozygotic (MZ), 85 male MZ, 72 female dizygotic (DZ), 15 male DZ, and 56 opposite-sex DZ pairs.

Among them, nearly half (184 MZ and 68 DZ) of the pairs had also participated in the on-campus cognitive test session in 2006–2011 and provided IQ data. Nearly 90% (334 MZ and 121 DZ) of the pairs had also responded to the postal survey administered in 2010–2011, which included the syllogism-solving test and provided syllogistic inference ability data.

The participants were informed of the purpose of the study, the research items, protection of their privacy, and their right to cancel their participation at any time. Signed informed consent was obtained for all participants at the beginning of each survey. All research procedures were approved by the ethics committee at the Faculty of Letters, Keio University.

### Measures

#### The Allais Problem

The two Allais problem questions, each of which consists of two options, were translated into Japanese. We calculated an approximately equivalent amount of money in Japanese currency and specified ¥100,000,000 instead of 1 million dollars. The participants were required to choose which option they preferred in the lottery between A and B, and then between C and D. It should be noted that the relatively smaller contribution of genetics to decision making compared to intelligence and personality is in part attributable to the larger component of measurement error, and that adjusting the measurement error by employing test–retest data would double the estimate for genetic effects (Beauchamp et al., 2011). To check the test–retest reliability of the Allais problem, we administered it twice with an interval of 4 months to 174 individuals from the sample. The concordance rate was 0.68, which allows us to estimate the variance for measurement error as 0.32. For individuals who had completed the problem twice, the first responses were used in the subsequent analyses.

#### Cognitive Ability Measures

We used IQ test scores and syllogism-solving test scores as cognitive ability measures. The IQ test was the full version of the Kyodai Nx 15- (Osaka and Umemoto, 1984; Lynn et al., 1987), one of the most popular group intelligence tests in Japan for participants over the age of 15. There are 12 subtests altogether. The individual scores of each subtest of the Kyodai Nx 15- were converted to standardized T scores, with a mean of 50 and standard deviation of 10, by following the score conversion chart attached to the scoring manual.

Following the principal component analysis with promax rotation conducted for all 12 subtests of the Kyodai Nx 15-, we used Verbal IQ and Spatial IQ indices (Shikishima et al., 2009). The Verbal IQ measure comprised the total standardized T score for the following four subtests: *Word completion*, *Sentence completion*, *Antonym or synonym*, and *Rearrangement of words*. The Spatial IQ measure comprised the total standardized T score for the following four subtests: *Punching a folded paper*, *Figure combination*, *Combination of figure and letter plates*, and *Sociogram.* The full IQ score with a mean of 100 and standard deviation of 15 was computed from total T scores of the 12 subtests, which also included *Memory*, *Matrix*, *Calculation method*, and *Index conversion* as well as Verbal and Spatial subtests, by following the IQ conversion chart. As we found a mean full IQ score of 101.66 and a standard deviation of 13.84, it was assumed that, in terms of the intelligence test scores, our twin sample was fairly representative of the general population.

The syllogism-solving test administered consisted of the five problems of the *BAROCO Short* (Shikishima et al., 2011). Syllogisms are a form of argument relating three terms that consist of two premises and a conclusion. Research indicates that syllogistic inference ability highly reflects a person’s general intelligence (Shikishima et al., 2009), and that performance on only five questions of the self-administered syllogism-solving test can be a useful index of general cognitive ability, which enables us to collect cognitive ability data more easily from more people compared with using IQ tests. The total score from the five problems, ranging from 0 points (all incorrect) to 5 points (all correct), was used in the analysis.

### Statistical Analysis

#### Univariate Genetic Analysis

We conducted univariate genetic analyses on each choice of the Allais problem response combinations. With the usual analysis on a continuous variable, the phenotypic variance (V*_P_*) of the variable can be partitioned into three variance components: additive genetic (V*_A_*), shared environmental (V*_C_*), and non-shared environmental (V*_E_*), which can be formulated as follows (Neale and Maes, 2002):

$$V_{p}=V_{A}+V_{C}+V_{E}$$

The additive genetic variance reflects variation in multiple genotypes whose influences are small and additive to form a quantitative phenotype. The shared environmental variance refers to the variation in environmental characteristics that makes family members alike and that differs from family to family. In contrast, the non-shared environmental variance reflects variation in environmental characteristics that makes family members different even if they live together. All the measurement error components are also included in the non-shared environmental variance.

The difference in genetic resemblance between MZ and DZ twin siblings (sharing 100 and 50%, respectively, of their segregating genes), and the equivalence in environmental resemblance for the two types of twins, can yield the following two equations concerning the observed covariances for MZ (Cov_MZ_) and DZ (Cov_DZ_) twin siblings:

$$C\,o\,v_{M\,Z}=V_{A}+V_{C}\;$$

$$C\,o\,v_{D\,Z}=0.5\,V_{A}+V_{C}\;$$

The estimates of the three parameters, V*_A_*, V*_C_*, and V*_E_*, can be computed by solving the above three equations.

In the present study, because the variable is binary, we applied an analytical method for categorical data (Rijsdijk and Sham, 2002) in the univariate genetic analysis. Total variance is constrained to unity, and the tetrachoric twin correlations using the threshold model (Cesarini et al., 2009a) are computed for both MZ and DZ twin pairs. The relative contribution of each parameter (i.e., *A*, *C*, and *E*) for each response combination of the Allais problem can be estimated by solving these coefficients (i.e., *a*^2^, *c*^2^, and *e*^2^) of the best fitting model with structural equation modeling using the maximum likelihood method (see Neale and Maes, 2002, for more details). The Mx software package was utilized for genetic analyses (Neale, 2004).

#### Bivariate Genetic Analysis

We conducted bivariate genetic analyses on each choice of the response combinations to the Allais problem and the cognitive test score to explore the extent to which underlying genetic and environmental factors mediate the phenotypic association, using Cholesky decompositions (Neale and Maes, 2002). With this technique, it is possible to estimate the relative degree to which the genetic effects on decision making overlap the genetic effects on cognitive ability, yielding a genetic correlation coefficient (*r*_G_). The shared environmental correlation (*r*_C_) and non-shared environmental correlation (*r*_E_) can be likewise obtained (for an explanation of *r*_G_, *r*_C_, and *r*_E_, see Neale and Maes, 2002). In the present study, because the decision-making data were binary and the cognitive ability data were continuous, polyserial correlations for both MZ and DZ twin pairs were used in the bivariate genetic analyses.

We highlighted the estimates whose 95% confidence intervals did not include zero (confidence intervals including zero indicate equivocal results, because they could signify small correlations or large standard errors as a result of the small variance components).

## Results

### Basic Statistics

The distribution of the responses for each of the two Allais problem questions is shown in **Table 1**. For the first question, twice as many respondents preferred certainty (A) to expected utility (B; 67% vs. 33%). For the second question, three times as many respondents preferred expected utility (D) to preferred certainty (C; 76% vs. 24%).

**Table 1:** Cross tabulation of the two Allais problem responses.

		Second question		Total
		C	D	
First question	A	215	593	808
	% of total	18%	49%	67%
	B	74	317	391
	% of total	6%	26%	33%
Total	Count	289	910	1199
	% of total	24%	76%	100%

For the combination of the two responses, the most frequently observed pattern, though not quite a majority choice, was AD (49%), which violates the EUT. The Allais paradox was therefore reproduced in the present study. The second most frequent pattern was BD (26%), which accords with the EUT. Fewer participants consistently preferred certainty (AC; 18%) and those who chose randomly (BC) were the smallest group (6%).

### Univariate Genetic Analysis

The model-fitting analysis of the threshold model revealed that for both response combinations of AD and BD the threshold between the response categories (i.e., the choice of AD or non-AD and BD or non-BD, respectively) should be set equal across twin sibling order and type of twin (MZ and DZ; **Table 2**). The response combinations of AC and BC were excluded from the genetic analysis due to insufficient number of cases.

**Table 2:** Model fitting for the response combinations AD/non-AD and BD/non-BD.

Model		-2LL	*df*	AIC	*Δχ2*	*Δdf*	*p*	ΔAIC
**Response combination of AD/non-D**
Saturated	ACE	1650.49	193	-735.51				
Equal threshold between order 1 and 2, between MZ and DZ	ACE	1653.09	1196	-738.91	2.60	3	0.46	-3.41
	**AE**	**1653.09**	**1197**	-**740.91**	2.60	**4**	**0.63**	-**5.41**
	CE	1654.84	1197	-739.16	4.35	4	0.36	-3.66
	E	1662.03	1198	-733.97	11.54	5	0.04	1.53
**Response combination of BD/non-BD**
Saturated	ACE	1365.42	1193	-1020.58				
Equal threshold between order 1 and 2, between MZ and DZ	ACE	1368.82	1196	-1023.18	3.40	3	0.33	-2.60
	**AE**	**1368.82**	**1197**	-**1025.18**	**3.40**	**4**	**0.49**	-**4.60**
	CE	1371.27	1197	-1022.73	5.85	4	0.21	-2.15
	E	1385.07	1198	-1010.93	19.65	5	0.001	9.65

*All x^2^ difference testing compares the fit of the Saturated Model to subsequent models; -2LL, log likelihood fit statistic; *df*, degrees of freedom; AIC, Akaike information criterion. A smaller value of AIC indicates a better fit to data. The best-fitting model is shown in bold*.

The tetrachoric twin correlation for the response combination choice AD was 0.24 for MZ twins and 0.12 for DZ twins. For the response combination BD, it was 0.35 for MZ twins and 0.18 for DZ twins. The AE model, in which the effect of shared environment (C) is not hypothesized, fitted best among all the models tested for both AD and BD.

The relative proportions of genetics (A) and non-shared environment (E) were estimated to be 23% and 77% for the choice of AD and 36 and 64% for the choice of BD, respectively (**Table 3**). If we take into account the measurement error estimated from the Allais problem test–retest reliability, for AD more than a half and for BD more than two thirds of the variance of the true score for the Allais problem can be inferred as genetic (for the correction of the contribution from the test–retest reliability, see Plomin et al., 2001).

**Table 3:** Parameter estimates for the best-fitting model.

Category	Threshold	*r*_MZ_	*r*_DZ_	Genetic (A)	Shared environmental (C)	Non-shared environmental (E)
AD/non-AD	0.01	0.24	0.12	0.23	–	0.77
				(0.08, 0.37)		(0.63, 0.92)
BD/non-BD	0.62	0.36	0.18	0.36	–	0.64
				(0.19, 0.51)		(0.49, 0.81)

*95% confidence intervals are shown in parentheses*.

### Mean Comparisons

**FIGURE 1:**
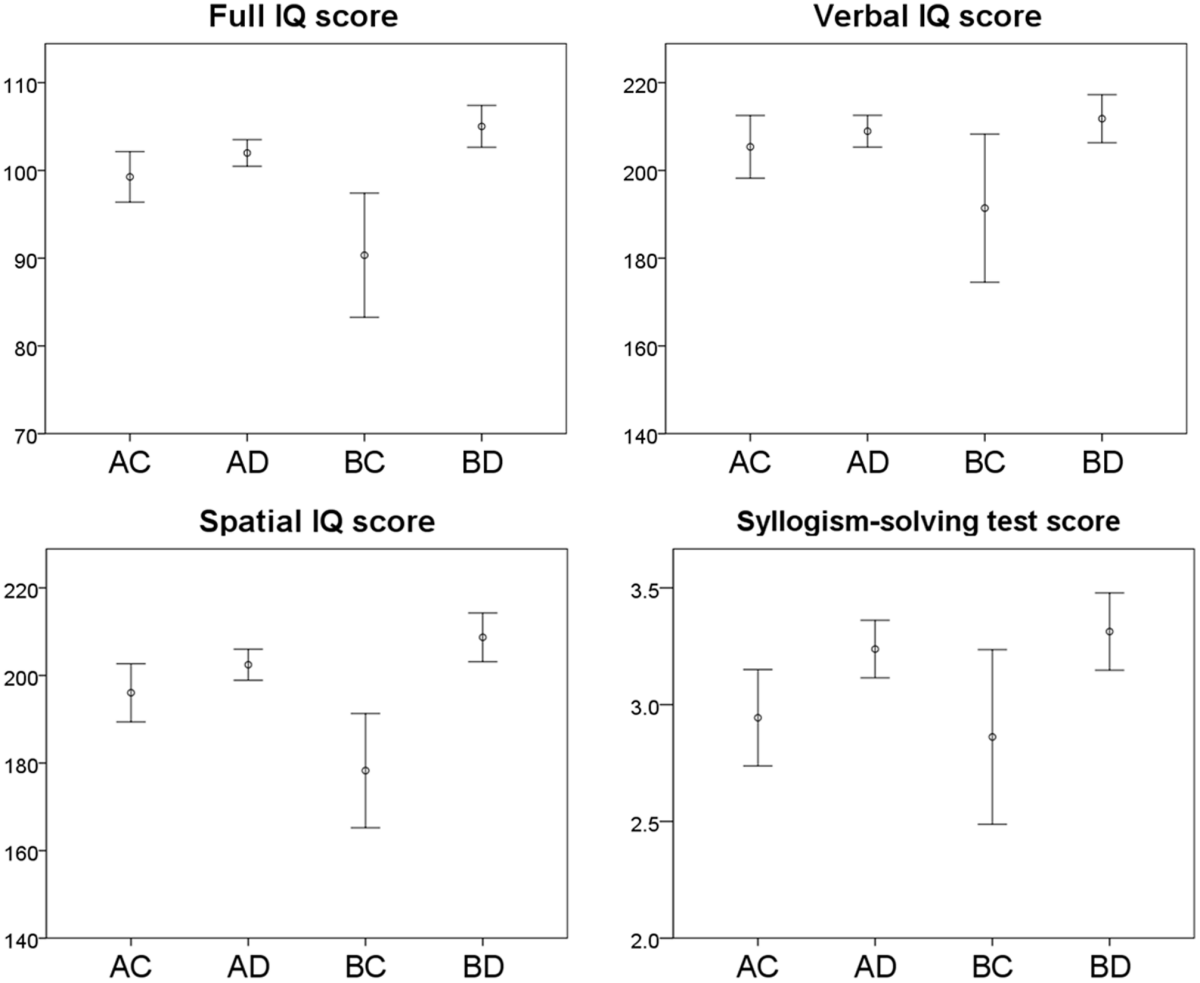
**Cognitive test scores and Allais problem response combinations. Error bars represent the 95% confidence intervals**.

Associations between Allais problem response combinations and full IQ, Verbal IQ, and Spatial IQ scores are shown graphically in **Figure 1**. The one-way ANOVA mean comparison tests of full IQ, Verbal IQ, and Spatial IQ scores among the four response combinations were all significant (*p* < 0.001, *p* < 0.05, and *p* < 0.001, respectively). Subsequent multiple comparisons with Bonferroni corrections showed that full IQ and Spatial IQ scores for those who chose BD, which reflects the EUT, were significantly higher than for those who chose AC (*p* < 0.05) and BC (*p* < 0.001), but the difference was not significant for AD. However, full IQ scores for those who chose BC were significantly lower than for those who chose other combinations (*p* < 0.05 for AC, *p* < 0.001 for AD, and *p* < 0.001 for BD).

The comparison of Verbal IQ scores showed that the scores for those who chose BD were significantly higher than for those who chose BC (*p* < 0.01), but the difference was not significant for AC and AD. The scores for those who chose BC were significantly lower than for those who chose AD (*p* < 0.05) and BD (*p* < 0.01).

Associations between the Allais problem response combinations and syllogism-solving test scores are shown in **Figure 1**. With an increased number of cases, the pattern was the same as that for IQ scores. The one-way ANOVA mean comparison test among the four response combinations was significant (*p* < 0.01). The multiple comparisons with Bonferroni corrections showed that scores for those who chose BD were significantly higher than for those who chose AC (*p* < 0.05).

### Bivariate Genetic Analysis

Bivariate genetic analyses of the relationship between the Allais problem response combinations and the four cognitive ability measures revealed that the choice of BD was significantly correlated with full IQ (*r* = 0.34), Spatial IQ (*r* = 0.57), and syllogistic inference ability (*r* = 0.28) at the genetic factor level, but no correlation was found at the environmental factor level (**Table 4**). The choice of the AD response combination indicated that there were no associations with cognitive ability measures at the genetic or environmental factor levels.

**Table 4:** Genetic and non-shared environmental correlations between response combination and cognitive ability.

	Genetic	Non-shared environmental
AD/non-AD and full IQ	0.07 (-0.21, 0.37)	-0.01 (-0.15, 0.13)
AD/non-AD and verbal IQ	0.06 (-0.22, 0.38)	0.02 (-0.12, 0.15)
AD/non-AD and spatial IQ	-0.08 (-0.41, 0.21)	0.11 (-0.03, 0.25)
AD/non-AD and syllogism solving	0.22 (-0.05, 0.53)	-0.01 (-0.11, 0.09)
BD/non-BD and full IQ	**0.34 (0.13, 0.56)**	0.07 (-0.07, 0.21)
BD/non-BD and verbal IQ	0.14 (-0.08, 0.36)	0.06 (-0.08, 0.20)
BD/non-BD and spatial IQ	**0.57 (0.34, 0.85)**	-0.12 (-0.26, 0.02)
BD/non-BD and syllogism solving	**0.28 (0.07, 0.49)**	-0.03 (-0.13, 0.07)

*95% confidence intervals are shown in parentheses. Coefficients whose confidence intervals did not include zero are shown in bold*.

## Discussion

We replicated the Allais paradox in a large Japanese twin sample with wide variation in cognitive ability. The relative frequency distribution of each response pattern was generally similar to those reported in Western populations (Conlisk, 1989; Huck and Müller, 2012). That is, we found that the PT compatible AD choice was the most frequently chosen, followed by the EUT compatible BD choice. The AC and BC choices were in the minority.

Using the twin method, we revealed that the individual differences in the response pattern of the Allais problem were influenced by genetics. The heritability of the AD choice was estimated to be 23 and 36% for the BD choice. When the measurement error was taken into account, the heritability estimates increased to more than 50 and 60%, respectively. Yet, neither was perfectly genetic (100% heritable) and the remaining influences were explained by non-shared or random environmental factors. There were no shared environmental or family influences for either of the strategy choices. This result is in accord with the “three laws of behavioral genetics” (Turkheimer, 2000); that is, (i) all human behavioral traits are genetic, (ii) shared environmental effects are smaller than genetic effects, and (iii) non-shared environmental effects are substantial. Therefore, from a behavioral genetic perspective, the causes of individual differences in decision making in economic behavior are not exceptional; rather, they are standard for human behavioral traits.

The results also demonstrated that individuals’ choices were related to individual differences in cognitive ability (general intelligence or *g*). It was shown that those who made the BD choice, which accords with the EUT, achieved higher scores on the full IQ and spatial IQ than those who made AC or BC choices. This suggests a significant association between the EUT directed decision making and general cognitive ability. In addition, participants who made the PT compatible AD choice and who made the certainty effect compatible AC choice had higher IQ scores than those who made the BC choice which probably reflected random responses. This indicates that these non-EUT choices also require some level of general cognitive ability; in this sense, they can also be regarded as “reasonable” decision making.

The bivariate genetic analysis revealed that the relationship between general cognitive ability and the BD choice was genetically mediated. The BD choice also had significant genetic correlations with syllogistic inference ability. Put differently, the phenotypic association between the BD choice and cognitive abilities is partially explained by a genetic association. These results indicate that those who are more intelligent genetically tend to make decisions according to the EUT. The fact that the BD choice exhibited a significant genetic correlation with Spatial IQ but not with Verbal IQ suggests that the association between the two reflects a common non-verbal mechanism, which should be specified in future work. Furthermore, the genetic correlation *per se* does not explain any particular mechanism for how genes actually operate. It might simply reflect the environmental effect that correlates with a person’s genetics (e.g., genetically intelligent people tend to receive higher education, with more opportunity to learn the EUT). Further elaboration is needed to identify the role of genetics in this regard.

On the other hand, the AD choice, which was compatible with the PT, was not genetically related to cognitive ability. This suggests that the biological/genetic mechanism directing AD choice is different from that of the BD choice. Loomes and Sugden (1982) proposed regret theory as an alternative to the PT in explaining why choice of AD is so attractive in the Allais problem. They purported that an individual’s capacity to anticipate feelings of regret could be a significant factor in violating the EUT and thus contravening the axioms of EUT was in fact “rational.” In line with this, Bourgeois-Gironde (2010) refined this theory from clinical and neurobiological perspectives. In light of this argument, the genetic effect observed in choosing AD may reflect individual genetic differences in susceptibility to rational emotion such as regret.

Discussions on the individual differences in decision making used to focus on the simple question, “who is rational?” (Stanovich, 1999). However, the very definition of rationality is a target of intense discussion. In the current study, we employed the Allais problem, one of the most famous and classical economic decision-making tasks, with at least three types of responses, namely BD, AD, and AC, which can be regarded as rational depending on the theories and perspectives one takes. We found that those individuals who provided these “rational” responses had higher general cognitive ability than those who made seemingly random and inconsistent responses (BC). Interestingly, though, we found that only the BD response, compatible with the EUT, had significant genetic correlations with cognitive abilities. In this sense, it was indicated that those who are genetically and economically rational (that is, in the sense of EUT) are those who are genetically intelligent. Taking another perspective, however, the results showed that non-EUT type rationality could be explained by genetic factors other than intelligence. Therefore, it can also be said that those who are non-EUT rational are those who are genetically disposed with some non-cognitive abilities, the details of which are yet to be determined. As Stanovich and West (2008) have suggested, the answer to the question “who is rational” is complex, and the current study provides part of the reason for this complexity: this is possibly because, to a certain extent, each rationality holds its own genetic foundation.

## Author Contributions

CS designed the study, analyzed the data, and drafted the manuscript. KH interpreted the data and wrote the paper. SY supported the analysis and interpreted the data. JA and MO interpreted the data, wrote the paper, and supervised the project. All authors approved the final version of the paper for submission.

## Conflict of Interest Statement

The authors declare that the research was conducted in the absence of any commercial or financial relationships that could be construed as a potential conflict of interest.
